# Thiamine tetrahydrofurfuryl disulfide promotes voluntary activity through dopaminergic activation in the medial prefrontal cortex

**DOI:** 10.1038/s41598-018-28462-2

**Published:** 2018-07-11

**Authors:** Masato Saiki, Takashi Matsui, Mariko Soya, Tomomi Kashibe, Takeru Shima, Takeshi Shimizu, Takehiro Naruto, Takahito Kitayoshi, Kouji Akimoto, Shinji Ninomiya, Hideaki Soya

**Affiliations:** 1 Laboratory of Exercise Biochemistry and Neuroendocrinology, Faculty of Health and Sport Sciences, University of Tsukuba, Tsukuba, Ibaraki 305-8574 Japan; 2 Sports Neuroscience Division, Advanced Research Initiative for Human High Performance (ARIHHP), University of Tsukuba, Tsukuba, Ibaraki 305-8574 Japan; 3 Sports Research and Development Core, University of Tsukuba, Tsukuba, Ibaraki 305-8574 Japan; 4 Takeda Consumer Healthcare Company Limited, Chiyoda, Tokyo 100-0005 Japan

## Abstract

A physically active lifestyle is associated with better health in body and mind, and it is urgent that supporting agents for such lifestyles be developed. In rodents, voluntary locomotor activity as an active physical behavior may be mediated by dopaminergic neurons (DNs). Thiamine phosphate esters can stimulate DNs, and we thus hypothesized that thiamine tetrahydrofurfuryl disulfide (TTFD), a thiamine derivative, promotes locomotor activity *via* DNs in rats. Acute i.p. administration of TTFD enhanced rat locomotor activity in a normal cage. *In vivo* microdialysis revealed that TTFD-enhanced locomotor activity was synchronized with dopamine release in the medial prefrontal cortex (mPFC). Antagonism of the dopamine D1 receptor, but not D2 receptor, in the mPFC fully suppressed TTFD-enhanced locomotor activity. Finally, we found a TTFD dose-dependent increase in voluntary wheel running. Our findings demonstrate that DNs in the mPFC mediates TTFD-enhanced locomotor activity, suggesting the potential of TTFD to induce active physical behavior.

## Introduction

The higher the level of physical activity, the higher the levels of physical fitness. Higher levels of physical activity produce various physiological and psychological benefits^1,2^, while inactivity leads to a lack of vitality in the body and mind, making it a risk factor for lifestyle diseases, depression, and Alzheimer desease^3–5^. Globally, however, 23% of adults and 81% of adolescents do not meet the WHO Global Recommendations on Physical Activity for Health^6^, and over 50% of employed adults and over 80% of over weight adults do not have leisure-time physical activity^7,8^. Thus, measures to enhance motivation for physical activity are required in modern human society.

Motivated behaviors, including locomotion, feeding, glucose seeking, and learning and memory, are regulated by the dopaminergic neurons (DNs)^9–13^. The substantia nigra pars compacta (SNc) and ventral tegmental area (VTA) in the midbrain are the origin nucleus of DNs^14^, and, in particular, DNs from the VTA that project into the medial prefrontal cortex (mPFC) are involved in the reward system^15^. Amphetamine (AMPH) injection induces dose-dependent dopamine release in the mPFC and increases voluntary locomotor activity through the dopamine D1 receptor in rats^16–18^. These data suggest that DNs in the mPFC are a potential target of agents that induce motivation for physical activity.

Although we must be careful of addiction induced by drugs such as AMPH^19^, thiamine tetrahydrofurfuryl disulfide (TTFD), a popular thiamine derivative, is a potential agent for the activation of DNs without severe side effects. Thiamine deficiency causes the development of Wernicke’s encephalopathy^20^, hence thiamine plays an important role in the central nervous system. TTFD is more rapidly absorbed than thiamine and it is metabolized into thiamine and its phosphorylated esters, which are thiamine monophosphate (TMP), thiamine diphosphate (TDP), and thiamine triphosphate (TTP)^21,22^. Chronic administration of TTFD ameliorates exercise-induced fatigue likely through the effect of TDP as a coenzyme of pyruvate dehydrogenase of skeletal muscles in humans and rats^23,24^. Further, the local injection of TTP and TDP into the rat striatum increases dopamine release^25^, suggesting a possible role of TTFD on DNs in the brain. However, the effects of TTFD on the brain, particularly on the DNs in the mPFC, and voluntary locomotor activity remain unclear.

A recent study showed that benfotiamine (BFT), another thiamine derivative, decreases stress-induced anxiety behavior and GSK-3β activity in the PFC^26^. BFT also prevents stress-suppressed adult hippocampal neurogenesis in predator-stressed mice, independent of brain TDP levels^27,28^, suggesting the potential of thiamine derivatives as a psychopharmacological agents. TTFD has a similar bioavailability to BFT^29^, indicating the possibility for a role of TTFD in the brain. Therefore, we hypothesized that TTFD has important effects on the brain and contributes to the induction of physical activity *via* D1-receptor-mediated dopaminergic activity in the mPFC.

To test the present hypothesis, we employed a rat model of acute TTFD injection, voluntary locomotor activity detection with infrared radiation, and *in vivo* microdialysis. First, we investigated the effect of acute TTFD i.p. injection on voluntary activity in rats in a normal cage. Next, *in vivo* microdialysis revealed the dopamine dynamics in the mPFC with TTFD injection. Third, we examined the inhibitory effects of dopamine D1 and D2 receptors on voluntary activity after TTFD injection. Finally, we assessed the effect of acute TTFD i.p. injection on voluntary running distance in a wheel cage.

## Results

### TTFD biphasically increases voluntary locomotor activity

**Figure 1:**
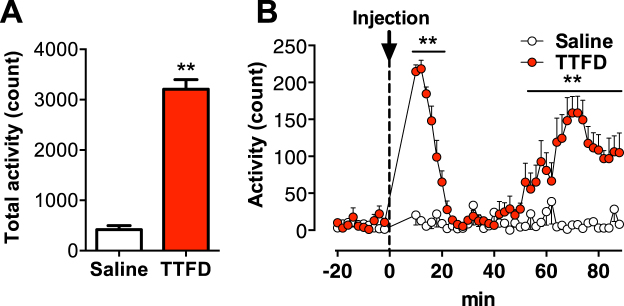
TTFD biphasically increases voluntary locomotion. Data are expressed as mean ± standard error (n = 5–9/group). (**A**) Total voluntary activity in a normal cage. **P < 0.01 versus saline group (unpaired t-test). (**B**) Voluntary activity in a normal cage for 120 min. *P < 0.05; **P < 0.01 versus saline group (two-way ANOVA with Bonferroni’s *post hoc* tests).

Rats were given i.p. injection of TTFD (50 mg/kg) or saline, and their voluntary activity in a normal cage were monitored for 90 min. TTFD increased the total voluntary activity for the entire 90 min (*P* < 0.01, Fig. 1A), and we found a biphasic enhancement of TTFD-induced voluntary activity at 10 to 20 min and 50 to 90 min after administration (*P* < 0.01, Fig. 1B). These results are the first evidence for TTFD as a potential agent for inducing voluntary locomotor activity.

### TTFD induces biphasic voluntary activity and dopamine release in the mPFC

**Figure 2:**
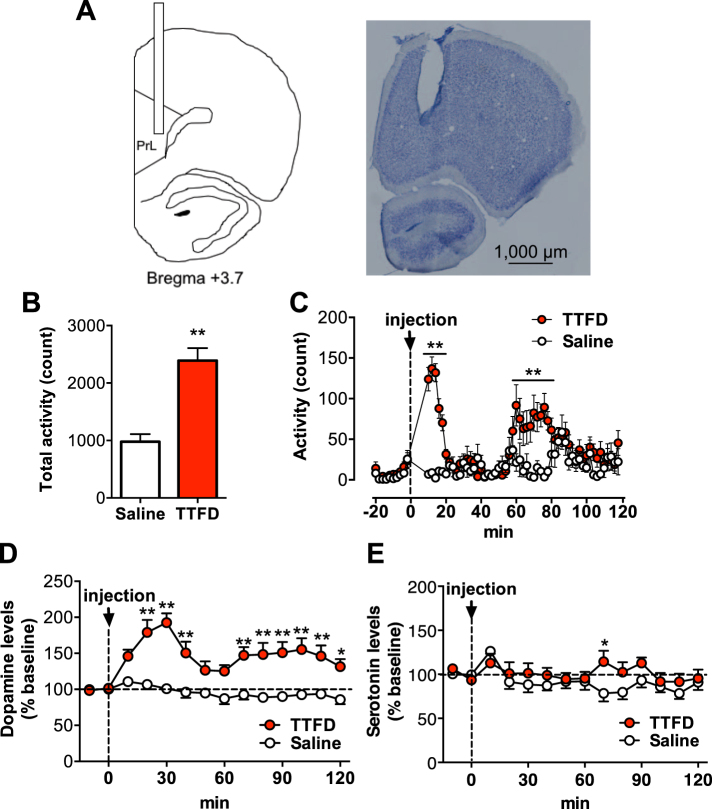
TTFD induces biphasic voluntary locomotion and dopamine release in the prelimbic cortex. Data are expressed as mean ± standard error (n = 11/group). (**A**) Schematic illustration and photomicrograph of brain section with Nissl staining. A guide cannula for microdialysis was implanted into the prelimbic area (PrL) of medial prefrontal cortex (mPFC). A scale bar represents 1,000 µm. (**B**) Total voluntary activity in a normal cage. **P < 0.01 versus saline group (unpaired t-test). (**C**) Voluntary activity in a normal cage for 120 min. (**D**) Extracellular dopamine levels. (**E**) Extracellular serotonin levels. *P < 0.05; **P < 0.01 versus saline group (two-way ANOVA with Bonferroni’s *post hoc* tests).

Rats were given an i.p. injection of TTFD (50 mg/kg) or saline, and the extracellular dopamine and serotonin levels in their mPFC were measured using *in vivo* microdialysis for 120 min while monitoring voluntary activity in a normal cage (Fig. 2A). Consistent with the first experiment, TTFD increased overall voluntary activity during the 120 min monitored (*P* < 0.01, Fig. 2B), and a biphasic enhancement of TTFD-induced voluntary activity was observed at 10 to 20 min and 60 to 80 min after administration (*P* < 0.01, Fig. 2C). Extracellular dopamine levels in the mPFC also increased biphasically at 20 to 40 min and 60 to 120 min after administration (*P* < 0.05, Fig. 2D). Serotonin levels remained basically unchanged but increased at 70 min after administration (*P* < 0.05, Fig. 2E). These results indicate the possibility that TTFD-enhanced voluntary activity is due to dopaminergic activation in the mPFC.

**Figure 3:**
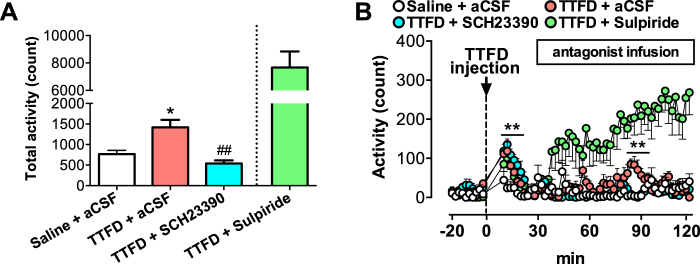
Dopamine D1 receptor antagonist, but not D2 receptor antagonist, inhibits TTFD-induced second peak of locomotor activation. Data are expressed as mean ± standard error (n = 5–6/group). (**A**) Total voluntary activity in a normal cage. *P < 0.05 versus saline + aCSF group, ^##^*P* < 0.01 versus TTFD + aCSF group (one-way ANOVA with Tukey’s *post hoc* tests). (**B**) Voluntary activity in a normal cage for 120 min. **P < 0.01 versus saline + aCSF group (two-way ANOVA with Bonferroni’s *post hoc* tests). The data from the TTFD + Sulpiride group are shown as a positive control and are excluded from statistical analysis by planned comparison.

### Antagonism of the dopamine D1 receptor, but not the D2 receptor, in the mPFC fully suppresses TTFD-induced voluntary activity

**Figure 4:**
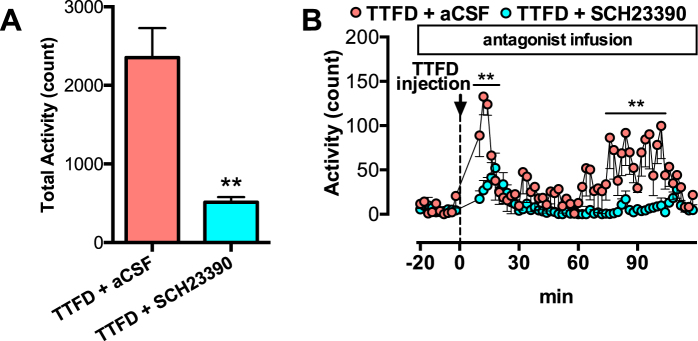
Dopamine D1 receptor antagonist inhibits TTFD-induced first peak of locomotor activation. Data are expressed as mean ± standard error (n = 6/group). (**A**) Total voluntary activity in a normal cage. **P < 0.01 versus TTFD + aCSF group (unpaired t-test). (**B**) Voluntary activity in a normal cage for 120 min. **P < 0.01 versus TTFD + aCSF group (two-way ANOVA with Bonferroni’s tests).

To examine whether TTFD induces voluntary activity through dopaminergic activation in the mPFC, we injected antagonists of the dopamine D1 and D2 receptors into the rat mPFC using microdialysis 30 min after TTFD administration. TTFD increased the total voluntary locomotor activity (*P* < 0.01), but the D1 receptor antagonist (SCH23390) fully inhibited the second peak of TTFD-induced voluntary activity (Fig. 3A). A D2 receptor antagonist (sulpiride) increased activity after injection (Fig. 3A,B), which is consistent with previous studies using D2-receptor antagonists^30,31^. In the present experiment, both antagonists increased dopamine and serotonin release in the mPFC (Fig. S1), indicating the validity of antagonist injection for central dopamine receptors^32^. We also injected SCH23390 into the mPFC 20 min before TTFD administration, and found that the antagonism of the D1 receptor fully inhibited both peaks of TTFD-induced voluntary locomotor activity (*P* < 0.01, Fig. 4A,B). These results directly support the present hypothesis that TTFD contributes to the induction of voluntary locomotor activity *via* D1-receptor-mediated dopaminergic activity in the mPFC.

### TTFD dose-dependent increases in voluntary running distance in running-wheel cage

**Figure 5:**
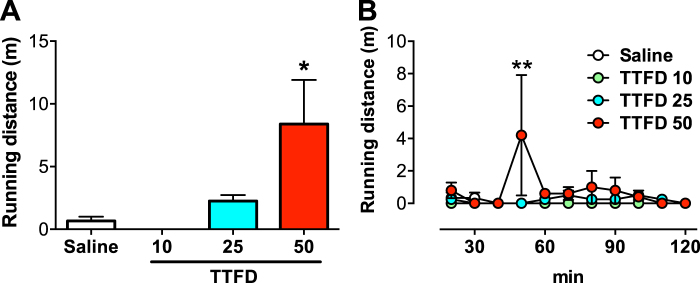
TTFD increases voluntary running distance in running-wheel cage. Data are expressed as mean ± standard error (n = 4–5/group). (**A**) Total running distance in a running-wheel cage. *P < 0.05 versus TTFD 10 mg/kg group (one-way ANOVA with Tukey’s *post hoc* tests). (**B**) Running distance in a running wheel cage for 120 min. **P < 0.01 versus saline group (two-way ANOVA with Bonferroni’s *post hoc* tests).

Finally, we examined whether acute TTFD administration increases not only voluntary locomotor activity but also the amount of running exercise using a running-wheel cage. The i.p. administration of TTFD increased voluntary running distance in a dose-dependent manner (*P* < 0.05, Fig. 5A), particularly at 40 to 50 min after administration (*P* < 0.01, Fig. 5B). These results imply that TTFD enhances not only voluntary locomotor activity but also running exercise distance.

## Discussion

This study tested the hypothesis that TTFD contributes to the induction of voluntary activity *via* D1-receptor-mediated dopaminergic activity in the mPFC. Our rat model of acute TTFD injection, voluntary activity detection with infrared radiation, and *in vivo* microdialysis showed that TTFD biphasically increases both voluntary activity and dopamine release in the mPFC (Figs 1 and 2). We also confirmed that antagonism of the dopamine D1 receptor, but not the D2 receptor, in the mPFC fully suppresses TTFD-induced voluntary activity (Figs 3 and 4). Furthermore, TTFD increased voluntary running distance in a dose-dependent manner in a wheel cage (Fig. 5). These findings support the present hypothesis and provide evidence for a possible role of TTFD in inducing physical activity.

Here, we determined the dose of the TTFD injection (50 mg/kg) based on a previous study that examined how TTFD counters physical fatigue by improving energy metabolism during a swimming exercise^23^. Previous studies investigating the effects of BFT on the brain employed 200 mg/kg^26,28^, which is four-fold higher than the dose of TTFD in the present study. However, we observed that a dose of 50 mg/kg of TTFD induces D1-receptor-mediated dopaminergic activity in the mPFC as well as voluntary activity (Figs 1–4). We also confirmed that TTFD-induced voluntary running occurred with a dose of 50 mg/kg, but not with lower doses (Fig. 5). These data suggest the validity of our TTFD injection model and that 50 mg/kg is likely the lower dose limit for observable effects of thiamine derivatives on rat brains.

TTFD induced biphasic voluntary locomotor activity at 10 to 20 min and 50 to 90 min after administration, which was synchronized with dopaminergic activity *via* the D1 receptor, but not with serotonin release, in the mPFC (Figs 1–4). This periodicity is consistent with the timing of the REM-nonREM sleep cycle in rats, which is about 10 min (7–13 min)^33^. Here we performed experiments during the light period, when rats normally sleep. Further, dopamine, rather than serotonin, plays an important role in the maintenance of an awake state *via* the D1 receptor^34,35^. Therefore, a periodicity of at least two or more occurrences of TTFD-induced voluntary activity might be due to an awake state regulated by dopaminergic activity in the REM-nonREM sleep cycle. However, why first peak is sharp and second peak is long-lasting must be investigated in the further research.

Our *in vivo* microdialysis revealed TTFD-induced dopamine release in the mPFC (Fig. 2D). DNs from the VTA (A10) project into the mPFC^14^, indicating that TTFD might activate DNs from the VTA. However, how TTFD activates DNs is still unclear. A possible mechanism is the effect of TTFD-derived TDP, a coenzyme of pyruvate dehydrogenase. Acetyl-CoA synthesized from pyruvate by pyruvate dehydrogenase is an important neuronal energy source derived from glucose or lactate, and can contribute to neuronal firing^36^. Furthermore, a previous study showed that local injection of TTP into the striatum increases dopamine release in the striatum, and that this was disrupted by an Na^+^ channel blocker (TTX), but not by a Ca^2+^ channel blocker (ω-CgTX)^25^. TTX can prevent the Na^+^ flux caused by TTP^37^. These previous studies indicate the possibility that TTFD increases dopamine release in the mPFC through energetic and signaling roles in enhancing Na^+^ permeability.

Furthermore, antagonism of the dopamine D1 receptor, but not the D2 receptor, in the mPFC fully suppressed the TTFD-induced voluntary locomotor activity (Figs 3 and 4). These results are consistent with previous studies showing that amphetamine increases dopamine release in the mPFC and locomotor activity of rats^16,17^, but that amphetamine-induced locomotor activity is inhibited by antagonism of the D1 receptor in the mPFC^18^. Also, we confirmed that antagonism of the D2 receptor induces a much higher level of locomotor activity (Fig. 3), which is consistent with previous studies showing hyper-behavior induced by D2-receptor antagonists through a disinhibiting effect^30,31^. Thus, we show the data with a D2-antagonist as a positive control, but this was excluded from statistical analyses because this was a planned comparison^38^. These findings support the present hypothesis that TTFD contributes to the induction of physical activity *via* D1-receptor-mediated dopaminergic activity in the mPFC, providing evidence for a possible role of TTFD in inducing physical activity.

We also observed that TTFD induces not only locomotor activity in rats in a normal cage (Figs 1–4), but also voluntary running in a dose-dependent manner in a running wheel cage at the timing following locomotor activation (Fig. 5). The degree of voluntary running behavior is regulated by DNs, likely *via* the D1 receptor in rodents^31,39,40^, suggesting a possible common neural mechanism in TTFD-induced locomotor activity and voluntary running. Although the linkage between TTFD-induced locomotor activity and wheel running is not fully investigated, TTFD-activated DNs together with locomotion could be a possible trigger for wheel running. Furthermore, chronic exercise, which is mimicked in rodents by voluntary running in a wheel cage^41,42^, produces various physiological and psychological benefits to prevent lifestyle diseases and to enhance brain functions in rodents and humans^43–46^. Although how TTFD-induced voluntary exercise affects physical and mental functions remains untested, TTFD may be a way to enhance active physical behavior with exercise and/or sport.

In general, motivation is the basis of animals’ behaviors such as food seeking, sexual behavior, and drug addiction relating with rewarding system^47^, which is regulated mainly by dopaminergic mechanisms^48,49^, and locomotion is a tool of it. However, previous studies showed that exercise itself activates dopamine metabolism in the brain (midbrain, striatum, hypothalamus, hippocampus etc.) likely for motor control and motivation in sustaining exercise, but it was returned to resting levels at fatigue^50–53^. Furthermore, dopaminergic mechanism could be involved in voluntary wheel running behavior in rodents^31,40^. These findings indicate the possibility for the presence of a motivation, intrinsic motivation, for exercise/locomotion itself. In the present study, TTFD increases locomotor activity *via* dopaminergic activation, independent of goals (Figs 1–4), suggesting a possible role for TTFD to promote motivation for locomotion and exercise.

TTFD is a popular agent for countering physical fatigue. Long-term administration of TTFD ameliorates the feeling of fatigue after exercise in trained participants^24^. Furthermore, six weeks of thiamine supplementation increases appetite, happiness, and decreases fatigue in elderly people with marginal thiamine deficiency^54^. These effects are likely exerted by TTFD/thiamine-derived TDP, a coenzyme of pyruvate dehydrogenase, which supports glucose metabolism in skeletal muscles^23^. In addition, in the current study, we observed for the first time that TTFD promotes dopamine release in the mPFC of normal healthy rats (Fig. 2D). Anti-depressant drugs, such as the dopamine reuptake inhibitor bupuropion, increase brain dopamine levels, producing psychological happiness in humans^55^ and preventing the onset of fatigue during prolonged exercise in rats^56^. Therefore, the anti-fatigue effect of TTFD could be induced not only by a metabolic effect, but also by dopaminergic activation in the brain.

Collectively, our findings provide direct evidence that TTFD administration induces voluntary locomotor activity *via* D1-receptor-mediated dopaminergic activity in the mPFC. TTFD also induces voluntary running in a dose-dependent manner, likely due to the same neural mechanism. This is the first study showing the effect of TTFD on the central nervous system. TTFD might help to promote physical activity, thereby improving physical and mental fitness.

## Materials and Methods

### Animals

Adult male Wister rats (SLC Inc., Shizuoka, Japan), housed and cared for in an animal facility, were fed a standard pellet diet (MF, Oriental Yeast Co., Ltd, Tokyo, Japan) and given water ad libitum. The room temperature was maintained at between 22 and 24 °C under a 12 h light/dark cycle (lights on: 07:00–19:00). All experimental protocols were approved by the Institutional Animal Care and Use Committee of the University of Tsukuba, and all procedures and methods were performed in accordance with the relevant guidelines laid down by the animal ethics committee (Animal ethical approval number: 17-066). Every effort was made to minimize the number of animals used as well as any pain and discomfort.

### Measurement of amount of voluntary activity

As per the method described by Lynch *et al*.^57^, the voluntary activity of rats was monitored in a cage (32 × 20 × 20 cm) using an Infrared Actimeter (Panlab, Barcelona, Spain). This device is equipped with three transparent cages, each with eight infrared lights located in a frame around the cage and connected to silent electronic counters. The apparatus is composed of a two-dimensional (horizontal and vertical axes) square frame, a frame support and a control unit. The lower tier records horizontal movements, while the upper tier records vertical movements. Samples were taken every 2 minutes. Raw data were computed with Actitrack® software (Panlab, Barcelona, Spain). Rats were fully acclimatized to the device for 30 minutes per day for 1 week. On the day of the test, after confirming that the rats in the device cages had been sedentary for 20 minutes, an i.p. injection of TTFD or a vehicle (saline) was administered. After administration, rats were placed back into the device cages and their movements were monitored. Each movement produced a signal caused by variation of inductance and capacity of the apparatus resonance circuit. These signals were automatically converted into numbers and locomotion was counted by number of samples where the position of the subject is different from its position during the previous sample and different to the position of the 2nd sample back in time. This was separated from emotional activity without position movement. The activity of each rat was automatically recorded for 120 minutes after TTFD administration.

### Surgery for microdialysis in the mPFC

The rats were anesthetized with isoflurane and placed in a stereotaxic instrument. An intracerebral guide cannula (outer diameter: 0.5 mm, AG-4, Eicom., Japan) was placed in the prelimbic area of the mPFC projected by DNs from VTA to regulate motivated behaviors^58,59^ (3.7 mm anterior to the bregma; 0.7 mm lateral; 3.0 mm below the pial surface)^60^. The cannula was secured to the skull with two anchoring screws and dental cement. To prevent occlusion, a dummy cannula (AD-4, Eicom., Japan) was inserted into the guide cannula. After the surgery, the animals were housed individually and were allowed to recover for at least week.

### Microdialysis for dopamine detection in the mPFC

A microdialysis probe was inserted into the mPFC *via* the implanted guide cannula connected and perfused with Ringer’s solution (147 mM NaCl, 4 mM KCl, and 2.3 mM CaCl_2_) at 2.0 μl/min so as to allow freely moving condition. A stable dialysate dopamine and serotonin concentration was usually obtained after a minimum of 2 h post-implantation of the probe. The 20 μl of microdialysate was collected in every 10 minutes using a fraction collector (EFC-82; Eicom, Japan), and then automatically injected into an HPLC-ECD system (HTEC-500; Eicom, Japan) by an autosampler (M-510; Eicom, Japan). Samples were analyzed for dopamine and serotonin concentration by the HPLC system with an EICOMPAK CAX column (2.0 mm,i.d. × 200 mm; Eicom, Japan) and a graft electrode (WE-3G; Eicom, Japan) set at 450 mV (vs Ag/AgCl reference electrode)^61^. The mobile phase contained 0.1 M ammonium acetate buffer, 0.05 mg/l sodium sulfate, 50 mg/l EDTA, and methanol (7:3, v/v), with a pH of 6.0. For data analysis, basal dopamine and serotonin concentration was estimated from an average of two HPLC time points before i.p. administration. At the end of each experiment, rats euthanized with pentobarbital, and the brain was removed. The position of the microdialysis probe was verified in coronal sections with Nissl staining.

### Microdialysis for antagonism of dopamine receptors in the mPFC

The dosages of antagonists used in this study were selected based on a previous study^2^. Rats were infused with a D1 antagonist (SCH23390; 10 mM) or a D2 antagonist (sulpiride; 10 mM) into the prelimbic area at a flow rate of 2 μl/min *via* the microdialysis probe. S-(−)-sulpiride was dissolved in 0.1 N acetic acid, then neutralized with 0.1 M NaHCO^3^ (pH 7.2) and brought up to volume. Other antagonists were dissolved in artificial cerebral spinal fluid (aCSF, 145 mM NaCl, 2.7 mM KCl, 1 mM MgCl_2_, 1.2 mM CaCl_2_, and 0.1 mM ascorbic acid). Before and during antagonism, we also collected dialysates, and these were analyzed for dopamine and serotonin concentrations using the HPLC system with a PP-ODS II column (4.6 × 30 mm; Eicom, Japan) and a graft electrode (WE-3G; Eicom, Japan) set at 400 mV (vs Ag/AgCl reference electrode). The mobile phase contained 0.1 M phosphate, 500 mg/l SDS, 50 mg/l EDTA and 2% v/v methanol, with a pH of 5.4^62^.

### Measurement of voluntary running distance

As per the method described by Lee *et al*.^41^, the voluntary running distance of rats was measured using a specially designed running-wheel apparatus (diameter = 31.8 cm, width = 10 cm; Rat Analyzer KI-103, Aptec, Kyoto, Japan). The resistance necessary to overcome the inertia of the wheel at its minimum load was 4.5 g. Distance is the number of revolutions times the circumference of the wheel. Rats were housed individually and had free access to the running-wheel apparatus for 1 week to be acclimatized. On the day of the experiment, the voluntary running distance of each rat was measured for 120 min.

### Statistical analysis

Data are expressed as mean ± standard error and analyzed using prism 5 (MDF Co., Ltd, Tokyo, Japan). Comparisons of the two groups were performed using Student’s t test for unpaired data. Group comparisons were performed using a one-way ANOVA, or two-way ANOVA with *post hoc* tests, including the planned comparisons^38^. Statistical significance was assumed at *P* < 0.05.

## Electronic supplementary material

Supplementary Information
