# Supplementary material for: Thiamine tetrahydrofurfuryl disulfide promotes voluntary activity through dopaminergic activation in the medial prefrontal cortex
Source: Sci Rep. 2018 Jul 11;8:10469. doi: 10.1038/s41598-018-28462-2 (PMC6041333; doi:10.1038/s41598-018-28462-2)
Supplement: Supplementary file 1 — Supplementary Information [file 41598_2018_28462_MOESM1_ESM.pdf]

Scientific Reports

Thiamine tetrahydrofurfuryl disulfide promotes voluntary activity through dopaminergic activation in the medial prefrontal cortex

Masato Saiki[1,#], Takashi Matsui[1,2,#], Mariko Soya[1,#], Tomomi Kashibe[1], Takeru Shima[1], Takeshi Shimizu[3], Takehiro Naruto[4], Takahito Kitayoshi[4], Kouji Akimoto[4], Shinji Ninomiya[4], and Hideaki Soya[1,2,*]

[1]Laboratory of Exercise Biochemistry and Neuroendocrinology, Faculty of Health and Sport Sciences, University of Tsukuba, Tsukuba, Ibaraki, Japan, 305-8574; [2] Sports Neuroscience Division, Advanced Research Initiative for Human High Performance (ARIHHP), University of Tsukuba; Tsukuba, Ibaraki, Japan, 305-8574; [3]Sports Research and Development Core, University of Tsukuba, Tsukuba, Ibaraki, Japan, 305-8574; [4]Takeda Consumer Healthcare Company Limited, Tokyo, Japan, 100-0005. [#]These authors contributed equally to this work.

***Correspondence**

Hideaki Soya, Ph.D.

E-mail: soya.hideaki.gt@u.tsukuba.ac.jp

Tel/Fax: +81-29-853-2620

Supplementary Information

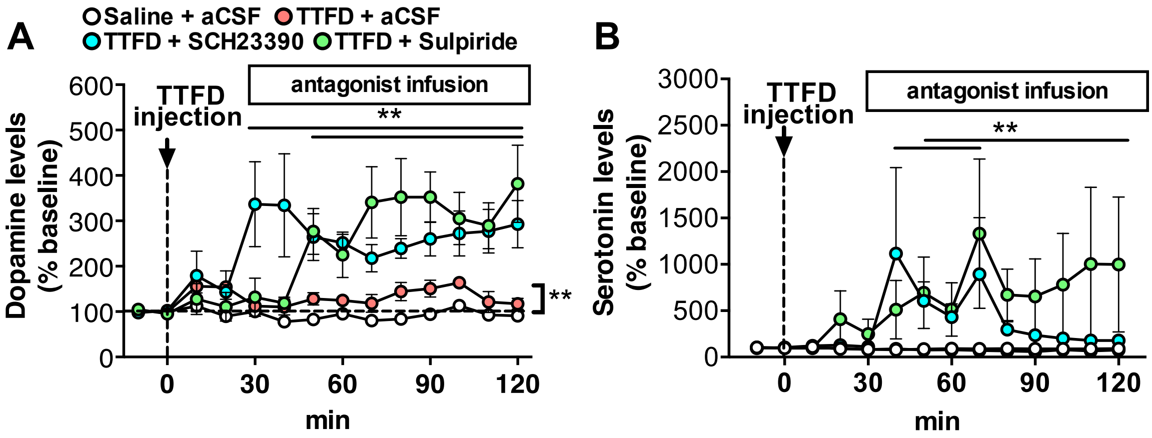

Figure S1 Dopamine D1 and D2 receptor antagonists increase dopamine and serotonin release after TTFD injection. (**A**) Extracellular dopamine levels. (**B**) Extracellular serotonin levels. $**P < 0.01$ versus saline + aCSF group (two-way ANOVA with Bonferroni's post hoc tests).
